# Perception of Undergraduate Medical Students on Elective Posting Block-2: A Relative Importance Index Approach

**DOI:** 10.7759/cureus.100802

**Published:** 2026-01-05

**Authors:** Mini Sharma, Monika Dengani, Sandeep Agrawal, Nirmal Verma, Kamlesh Jain

**Affiliations:** 1 Community Medicine, Pt. Jawahar Lal Nehru Memorial Medical College, Raipur, IND; 2 Community Medicine, Late Smt Indira Gandhi Memorial Government Medical College, Kanker, IND

**Keywords:** block-2, elective posting, perception, relative importance index, satisfaction

## Abstract

Background

The elective module is a new addition to the undergraduate medical curriculum in India, in which students choose the specialty on their own, where they are required to spend eight weeks. This study aimed to assess students’ perception regarding their elective posting in Block-2 through the use of the Relative Importance Index (RII).

Methodology

This cross-sectional study included undergraduate students. Data were collected from 181 participants by using an online structured Google Forms questionnaire. Students were asked to provide their perception on elective posting Block-2 on five domains, i.e., learning benefits, role of preceptor and teamwork, satisfaction with the elective, perceived electives, and negative response, and the statements were ranked through calculation using the RII.

Results

The RII revealed that the most important domains were the role of preceptors and teamwork (0.65), followed by negative response (0.52), satisfaction with electives (0.45), learning benefits (0.43), and perceived electives (0.42).

Conclusions

Students perceived the role of preceptor and teamwork as the most important domain. The success of elective posting lies with the effort of medical teachers to execute and implement in such a novel way that each student becomes a lifelong learner.

## Introduction

To emphasize the value of improving competency in professional skills and knowledge, a competency-based medical curriculum for Indian medical undergraduates has been implemented in India since 2019 by the National Medical Commission (NMC). The NMC introduced a two-month mandatory elective posting in the curriculum with an orientation toward clinical, laboratory sciences, and research work training, which helps students develop competence at all levels and helps produce a competent doctor [1-5]. In addition, it provides an opportunity to self-choose the specialty of their future interest within or outside the institution, so that exposure to the real world of medicine and practice would not create difficulty for them. Moreover, elective experience can help them decide their future specialization in the field of medicine [6,7]. The existing literature highlights the elective as a valuable course by delivering competent learning that may lead to academic achievement [6,8].

The success of the elective program depends upon its effective implementation at individual colleges, which relies on dedicated and requisite staff providing opportunity for learning in a supportive environment that allows students to maximize their potential [9].

Previous studies have reported that good academic activities for an appropriate duration and the helpful nature of faculty members encourage students to engage in interesting and meaningful training during elective periods with an opportunity to learn, which, in turn, contributes to training a competent doctor [7,10-13].

Understanding students’ perceptions is a tool to monitor program effectiveness, which enables us to refine our implementation policies and anticipate future program success. Students’ perception of experience and lessons learnt during their elective have been the subject of previous studies [7,9,14].

However, the educational benefit of an elective is mostly determined by its implementation at an institutional level. Several issues that notably affect students’ learning experience include clarity of objectives, assessment of learning resources, sufficient time allocation, illness-related absenteeism, quality mentorship, and modalities of assessment, as well as faculty involvement. In contrast, lack of supervision, poor communication, and discrepancies between the students’ expectations and the course content, together with administrative problems, have also been reported to negatively influence students’ elective learning experiences [10-12,15-17].

According to Khilnani et al. (2018), successful program implementation needs a good understanding of the subjects by the learners. Hence, for effective implementation, well-structured elective programs provide students with opportunities to learn with interest while facing some challenges [4,11]. Facing problems during elective programs can negatively impact students’ learning experience and overall outcome, yet the majority of students find their elective course worth the time and effort [8,11].

Competency in knowledge and skills produces outstanding undergraduates, which also helps establish their own learning process [4,9]. Hence, there is an urgent need to understand the perception of undergraduate medical students that influences the importance of elective training in the medical curriculum. In line with the importance of understanding elective training of medical undergraduates, this study aimed to measure and rank the perception of students toward elective posting by their level of importance using the Relative Importance Index (RII).

## Materials and methods

This descriptive, cross-sectional study was conducted at Pt. Jawaharlal Nehru Memorial (JNM) Medical College, Raipur, Chhattisgarh, India, over a six-month period from March 2023 to August 2023. The study population consisted of undergraduate medical students enrolled at the institution during the study period. Universal sampling was employed, whereby all eligible students were invited to participate. Eligibility was defined as completion of the elective posting (Block-2) during the study period. Consequently, all 181 undergraduate students who fulfilled the inclusion criteria were included in the study. The study received ethical approval from the Institutional Ethics Committee (approval number: No./MC/ETHICS/2023/402).

Data were collected using a 40-item, self-designed, semi-structured questionnaire, which was developed after an extensive literature review by research experts from the Department of Community Medicine [1]. The face and construct validity were ensured for the appropriateness, applicability, and non-repeatability of the questions. Finally, the questionnaire included 31 items on the perception of students regarding the elective module using confirmatory factor analysis [1]. The internal reliability using Cronbach’s alpha was 0.951, which indicates a high level of internal consistency (a value >0.70 is considered acceptable) for the scale with this specific sample.

A WhatsApp group of 181 study subjects was created, and the study questionnaire was shared via Google Forms and asked to be submitted after their consent. The response rate was 100% with no missing data. The tool was administered via Google Forms, chosen for its accessibility, cost-effectiveness, and efficiency in facilitating real-time, paperless data collection and analysis. Written consent was electronically obtained at the beginning of the Google Forms, in line with ethical standards for minimal-risk digital surveys. Participants were fully informed about the study’s purpose, confidentiality, voluntary participation, and their right to withdraw at any stage [18,19].

The questionnaire consisted of two sections: (a) demographic details, including age and gender; (b) perception of the Block-2 elective posting, assessed across the following five domains: (1) perceived electives, (2) learning benefits, (3) role of the preceptor and teamwork, (4) negative responses of students, and (5) satisfaction with the elective.

Students’ perceptions were measured using a five-point Likert scale ranging from 1 (strongly disagree) to 5 (strongly agree). Due to the presence of negatively worded items in the questionnaire, reverse coding of the Likert scale was done, which ranged from 1 (strongly agree) to 5 (strongly disagree) [20-22].

To analyze the importance of various factors, the RII was calculated using Microsoft Excel version 365. RII is particularly suitable for analyzing Likert-scale data and was computed using the following formula: RII = (ΣW)/(A × N), where W is the weight given to each item by respondents (1 to 5), A is the highest possible weight (5), and N is the total number of respondents. The RII values range from 0 to 1, and were categorized as follows [23,24] (Table 1).

**Table 1:** Interpretation of RII values along with importance index. RII = Relative Importance Index

RII value	Importance level	Interpretation
0.80–1.00	High	Strongly agree
0.60–0.80	High-medium	Agree
0.40–0.60	Medium	Neutral
0.20–0.40	Medium-low	Disagree
0.00–0.20	Low	Strongly disagree

The average RII at the domain level was calculated by taking the sum of the RII scores of each item divided by the total number of items under each domain (e.g., for students’ perception domain: average RII = (0.802 + 0.794 + 0.796 + 0.727)/4 = 0.78).

## Results

A total of 181 undergraduate students participated in the study, including 102 (56.4%) male and 79 (43.6%) female students. The mean age of the students was 22.96 ± 1.0 years. This domain assessed students’ perception of electives. The highest RII (0.802) was observed for clarity of electives’ objectives, which showed that before starting a course, a clear objective can be very useful for students’ learning experience. The clear objective of the course meets the expectations of the students. This was followed by RIIs of 0.796 and 0.794, which indicated students’ agreement on the usefulness of learning sources, which were used during elective posting, and sufficient time allocation was provided to understand the topic of the elective module, respectively. Hence, well-designed and diverse resources make learning possible and beneficial, with both reporting a high to medium importance level. It was also observed that time allocation was adequate for understanding the topic. Effective time allocation can impact students’ efficiency, productivity, and goal achievement. However, the lowest RII in this domain of 0.727 was noted with appropriateness of the current assessment components of elective, which showed a high to medium importance level (Table 2).

**Table 2:** Ranking of students’ perception of the elective during elective posting by using RII. RII = Relative Importance Index

Statement	Response score					Mean	RII	Rank	Level of importance
	Strongly disagree, 1	Disagree, 2	Neutral, 3	Agree, 4	Strongly agree, 5				
The objectives of the elective were clear	3	3	20	118	37	1.99	0.802	1	High
Time allocation was adequate to understand the topic	2	3	30	109	37	2.03	0.794	3	High-medium
The learning sources were useful	0	3	32	112	34	2.02	0.796	2	High-medium
Appropriate assessment of the components	7	4	51	105	14	2.36	0.727	4	High-medium

All of the variables of learning benefits were perceived to be of a high to medium importance level. The RII score ranged from 0.746 to 0.797. The highest RII of 0.797 was given for learning new information during the elective program, followed by opportunities for hands-on clinical work (RII = 0.794) and improvement in practical skills (RII = 0.782). The lowest-ranked item was students’ confidence after the elective (Table 3).

**Table 3:** Ranking of learning benefits of the elective during elective posting by using RII. RII = Relative Importance Index

Statement	Response score					Mean	RII	Rank	Level of importance
	Strongly disagree, 1	Disagree, 2	Neutral, 3	Agree, 4	Strongly agree, 5				
In-depth theoretical knowledge was gained	4	3	36	104	34	2.11	0.778	6	High-medium
In-depth practical knowledge was gained	1	5	36	109	30	2.10	0.779	5	High-medium
Improvement in practical skills	2	3	35	110	31	2.09	0.782	3	High-medium
Knowledge and skills acquired will help in clinical practice	0	4	32	122	23	2.09	0.781	4	High-medium
Encouraged intellectual curiosity	3	3	34	122	19	2.17	0.767	8	High-medium
Better understanding of difficult topics	2	3	41	112	23	2.17	0.767	7	High-medium
Opportunity to explore the topic	4	4	44	109	20	2.24	0.751	9	High-medium
More confident in my ability after electives	5	4	46	106	20	2.27	0.746	11	High-medium
Learnt useful and novel information	1	1	29	119	31	2.02	0.797	1	High-medium
Helped in choosing the future specialty	6	5	46	97	27	2.26	0.748	10	High-medium
Gave opportunity for hands-on clinical work	1	3	25	123	29	2.03	0.794	2	High-medium

In an elective posting, both the preceptor’s role and teamwork are crucial for a positive learning experience. In this domain, the highest RII of 0.806 was given to collaborative learning, which enhanced problem-solving. This was followed by RII scores of 0.799 and 0.775, which indicated that the preceptor fostered students to think and perform work independently in electives while providing full attention and help during the elective module, respectively. However, the lowest rank was noted for the incorporation of extra efforts of the preceptor (RII = 0.750) into these elective modules relative to regular courses. This indicates that students take the elective as an opportunity to improve their knowledge, skills, and teamwork (Table 4).

**Table 4:** Ranking of the role of preceptors and team work during elective posting by using RII. RII = Relative Importance Index

Statement	Response score					Mean	RII	Rank	Level of importance
	Strongly disagree, 1	Disagree, 2	Neutral, 3	Agree, 4	Strongly agree, 5				
Encouraged to perform independently	2	1	29	113	36	2.01	0.799	2	High-medium
Helped and paid attention	1	3	44	103	30	2.13	0.775	3	High-medium
Incorporated extra efforts	3	3	55	95	25	2.25	0.750	4	High-medium
Made working in a team easy	0	4	27	110	40	1.99	0.806	1	High

Negative responses refer to situations where the elective course did not meet the expectations of students, leading to a negative experience or feedback. The RII in this domain ranged from 0.571 to 0.745, which showed that the majority of the students disliked the elective course because faculties were less helpful in explaining the topic (RII = 0.745). Further, students felt that learning through an elective course was ineffective and insufficient (RII = 0.708), followed by the elective course being a waste of time (RII = 0.692). Elective course requiring extra work or efforts was considered the least Important variable (RII = 0.571). It may indicate that students found the elective course uninteresting, which led to their reduced learning (Table 5).

**Table 5:** Ranking of negative responses during elective posting by using RII. RII = Relative Importance Index

Statement	Response score					Mean	RII	Rank	Level of importance
	Strongly disagree, 1	Disagree, 2	Neutral, 3	Agree, 4	Strongly agree, 5				
Was a waste of time	10	29	54	44	44	2.54	0.692	3	High-medium
Was ineffective and insufficient	7	27	54	47	46	2.46	0.708	2	High-medium
Faculty were less helpful	6	23	45	48	59	2.28	0.745	1	High-medium
Required extra work and efforts	11	55	74	31	10	3.14	0.571	4	Medium

The majority of the respondents expressed satisfaction with the elective course, which sparked new interest in them, with the highest RII of 0.787, followed by the RII of 0.769 (satisfied with the content) and 0.757 (satisfactory method of assessment), showing a high to medium importance index. However, they ranked their experience of the elective as fulfilling their expectation as the least important variable (RII = 0.736) (Table 6).

**Table 6:** Ranking of satisfied with the elective during elective posting by using RII. RII = Relative Importance Index

Statement	Response score					Mean	RII	Rank	Level of importance
	Strongly disagree, 1	Disagree, 2	Neutral, 3	Agree, 4	Strongly agree, 5				
Satisfactory method of assessment	0	1	59	99	22	2.22	0.757	3	High-medium
Satisfaction with elective modules	7	6	36	110	22	2.26	0.748	4	High-medium
Satisfied with the content	4	4	35	111	27	2.15	0.769	2	High-medium
Helpful in exploring interesting areas	3	2	25	125	26	2.07	0.787	1	High-medium
Satisfactory workload	4	2	50	107	18	2.27	0.747	5	High-medium
Fulfilled course expectations	7	5	49	98	22	2.32	0.736	8	High-medium
Satisfactory method of allotment	9	8	38	102	24	2.31	0.737	7	High-medium
The course was beneficial	6	3	48	103	21	2.28	0.744	6	High-medium

**Figure 1:**
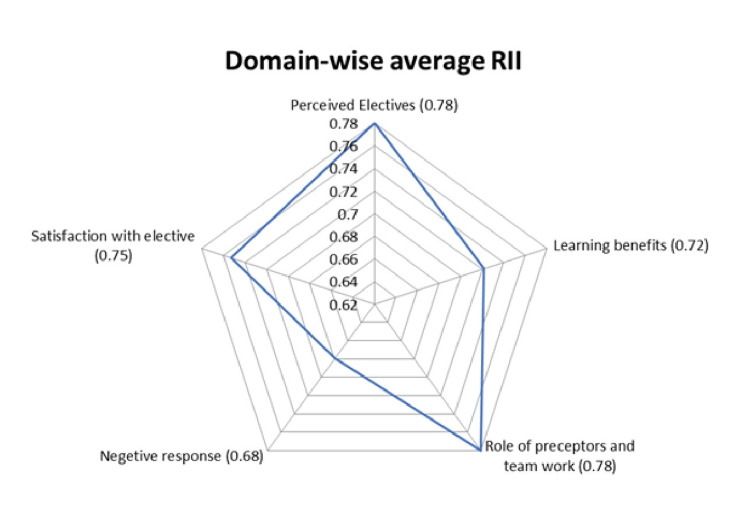
Spider chart representing average RII of the five domains of the elective course. RII = Relative Importance Index

The average score of five domains showed the highest score for perceived elective (0.78) and role of preceptors and teamwork (0.78), indicating the importance of the elective course as high to medium when knowledge and skill are gained as a team with active mentorship. The lowest average RII was noted for negative response (0.68), suggesting that the course needs to improve its focus on learning sources, time allocation, and faculty efforts. Learning benefits (0.72) and satisfaction with electives (0.75) also had high to medium average RII scores, indicating positives such as learning new information and practical skills can be achieved by continuing such elective module learning for the students (Figure 1).

## Discussion

This study introduced the ranking of perception of undergraduate medical students regarding elective courses by using the RII. The clear objective of an elective suggests that it can influence students’ understanding regarding course expectations before they begin, because at the start of the course, when students know what they are getting into, it creates an environment where they likely feel more confident, leading students to focus on learning rather than worrying or having anxiety about the future [15,25]. Furthermore, useful learning resources with sufficient time allocation during an elective course strengthen understanding and retention [10]. Another strong positive response was learning new information, which allows them to develop specialized skills [17].

Although students were able to learn individually, learning with other students as a team helps them to achieve a common goal, which might be difficult to achieve alone, as well as creates opportunities for both students and faculty [10].

The respondents also reported that the less supportive involvement of the faculty during the elective course was the main factor leading to a negative perception. This finding is in contrast to another study [10]. Furthermore, less involvement of the faculty decreased their interest, and they felt their course to be a waste of time and ineffective. Therefore, increased supportive involvement of the faculty would help align the expectations of students with actual course content, reducing disappointment or confusion. Therefore, for the smooth implementation of medical education, there is a need for a faculty development program for improving them as competent teachers and trainers [26].

The high RII score in this study has shown that elective courses are incredibly valuable to discover or deepen existing interests, with a chance to develop new knowledge and skills. Students’ satisfaction is a relatively complex concept that depends on the balance between students’ expectations and their perceived experience of learning.

The analysis of relative importance also indicated that the most important domain that influences the perception of elective courses among students was the role of preceptor and teamwork, which is crucial for the successful implementation of the elective course. The role of a preceptor should be considered a critical component for the holistic development of students.

The limitation of the study was that it was conducted at a single institution, resulting in a limited sample size. Therefore, the findings essentially represent the views of students from this particular institution only. Thus, the results from this study cannot be generalized to all medical colleges of the state or the entire country.

## Conclusions

This study provides an important insight into undergraduate medical students’ perception regarding elective posting Block-2 by using RII to extensively evaluate the different aspects of the elective program. The study findings suggested that students perceived the role of preceptor and teamwork and perceived electives as the most important domain, followed by satisfaction with electives, learning benefits, and negative response. Through RII analysis, all the criteria were ranked as high, high-medium, or medium in importance in the study. The overall RII analysis revealed that the two statements “objectives of the elective were clear” and “electives made ease in working in a team” had a high importance level. Overall, the study highlights the perception of students, which is critical to assess the shortcomings and benefits of elective courses. The study has shown that elective courses are incredibly valuable to discover or deepen existing interests, with an opportunity to develop new knowledge and skills. Students’ satisfaction is a complex concept that depends on the balance between students’ expectations and the perceived experience of learning.

## Appendices

**Table 7:** Elective module questionnaire. *: Reverse coding. Strongly disagree = 1, disagree = 2, neutral = 3, agree = 4, strongly agree = 5.

Serial number	Domain	Questionnaire	1	2	3	4	5
1	Perceived electives	Were the objectives of the electives clear to me in advance/beforehand?					
2		Was the time allocation adequate to understand the chosen elective?					
3		Were the learning resources useful?					
4		Were the current assessment components in the elective appropriate?					
5	Learning benefits	I gained in-depth theoretical knowledge about the chosen elective					
6		I gained in-depth practical knowledge about the chosen elective					
7		I noticed improvement in my practical skills					
8		Knowledge and skills acquired through the elective will help me in clinical practice					
9		The elective encouraged my intellectual curiosity					
10		Elective helps me to better understand the difficult topics easily					
11		I was provided with the opportunity to explore the topic independently					
12		I feel more confident in my ability after the elective posting					
13		I learned new information during the elective, which was useful to me					
14		The elective made me feel more prepared in choosing my future specialty					
15		The elective provided me with adequate opportunities for hands-on clinical work					
16	Role of preceptors and teamwork	My preceptor succeeded in encouraging me to think and perform independently					
17		My preceptor paid enough attention and helped during the elective module					
18		My preceptor put more effort into this elective module compared to regular courses					
19		Learning with other students as a team increased my ability to understand and solve clinical or patient problems together					
20	Negative perception*	I dislike elective modules; it is a waste of time					
21		I feel learning through elective modules is ineffective					
22		I dislike elective modules because faculties are less helpful in understanding the topic					
23		Elective modules require too much extra work or efforts					
24	Satisfaction with the elective	I was satisfied with the elective modules					
25		I was satisfied with the content of my elective topic					
26		Electives are helpful in exploring interesting areas					
27		The workload in the elective was satisfactory/acceptable					
28		The elective modules met my expectations					
29		Overall, the elective modules were very beneficial for me					
30		Was the allotment method of electives satisfactory					
31		Was the assessment method of electives satisfactory					
